# The decline of clinical skills: a challenge for medical schools

**DOI:** 10.5116/ijme.5b3f.9fb3

**Published:** 2018-07-13

**Authors:** Fabrizia Faustinella, Robin J. Jacobs

**Affiliations:** 1 Baylor College of Medicine, Department of Family and Community Medicine, USA

**Keywords:** Clinical skills, challenge, medical schools, USA

## Introduction

There is a consensus that physical examination skills have been greatly deteriorating during the past twenty years,^1^^-^^4^ with some reports dating back to the 1970s.^5^ For this reason, medical schools and residency programs have increased the development of courses, workshops and symposia with the specific goal to improve the quality and value of the physical exam. The problem of deteriorating physical examination skills has gained worldwide attention through an increase in publications and discussion panels.^6^^-^^9^

While there is an increasing focus on the decline of effective approaches to the physical exam, few medical educators are teaching comprehensive clinical skills, including history taking skills, which is a critical aspect of good medical practice. The medical interview remains “the most powerful and sensitive and most versatile instrument available to the physician.”^10^ Inaccurate and incomplete patient histories are among the leading causes for diagnostic errors.^11^^,^^12^ As a course director of a Physical Diagnosis Course and the Comprehensive Clinical Competencies Examination and educator of bedside medicine, I have witnessed poor interviewing skills by students, which are key to problem detection and accuracy of diagnoses. Reports indicate that physicians are able to collect 60% to 80% of the information relevant for a diagnosis just by taking a medical history,^13^^-^^16^ leading to a final diagnosis in more than 70% of cases.^13^ Moreover, effective communication during the medical interview is critical to the formulation of a positive physician-patient relationship, which can result in better health outcomes.

It, therefore, behooves academic medical institutions to examine the possible causes leading to the decline of specific clinical skills such as history taking and physical examination to mitigate the problem. The three clinical cases presented below illustrate how the lack of proper history taking resulted in a poorly performed physical exam with observable negative consequences for the patient.

### The Lost Art of the Physical Examination

Turn on the light!  During rounds, a case was presented as ‘fever of unknown origin.’ The patient, an elderly Hispanic woman with no English language skills, was the final admission of the night shift, arriving at the medicine floor at 5.30 am. At 7:00 a.m. I got to the patient’s room, which was dark, and after introducing myself, I asked her if it would be all right if I turned on the lights. I asked her (in Spanish) the most conventional of questions: “What happened? Why did you come to the emergency department?” The patient answered, "because my right leg is hurting." I removed the bed covers to find that her leg had observable indications of cellulitis. I asked the resident and the student assigned to the case how they came to ascertain the cause of the fever was unknown. They responded, “We didn't turn the light on when we examined the patient.”  At that point, I wondered if they had even examined the patient at all, or if they ever asked her about her symptoms. This case highlights impediments in several capacities, such as poor communication on behalf of the emergency department (ED) staff, the language barriers, and insufficient or non-existent history taking. Other possible reasons include patient overload, scattered multitasking, time constraints, and lack of available interpreters. These insufficiencies in effective physical examination and history taking skills lead to a delayed diagnosis and initiation of therapy.

Doctor, I am NOT having a heart attack! A 48-year-old man came to the clinic complaining of severe chest pain. I witnessed the resident, before examining the patient or taking a history and without hesitation, order an electrocardiogram (ECG). Without intervening, as I was in the midst of a phone consult with a colleague, I watched the situation unfold. Shortly after, the nurse exited the room with a normal ECG in her hands, commenting about the terrible skin rash on the patient’s chest wall. Ultimately, the actual cause of the chest pain was a case of Herpes Zoster. The ECG could have been completely avoided, had the patient been asked questions and methodically examined.

Talk to the patient! A 24-year-old Burmese woman with no English language skills came to the clinic. A phone interpreter was contacted to translate. The patient, who had given birth to a healthy baby three weeks prior to presentation, reported sudden onset of vaginal pain, dysuria, and stated that something was “coming out”.  The physical exam revealed the presence of a vaginal mass, most likely consistent with uterine prolapse. Because she was unable to obtain a timely appointment with a gynecologist, the patient was sent with a written referral – including the diagnosis – to the local county hospital emergency department. Later when I reviewed the patient’s electronic medical record, it indicated she was to be discharged from the ED with a diagnosis of urinary tract infection. I immediately called the ED to talk to the attending physician who told me that, most likely, the resident, busy running around, had not even talked to the patient. Therefore, there was no history taking or physical examination. This lack of attention to the patient led to an incorrect diagnosis, despite my written referral that nobody read.

These cases illustrate how poor, insufficient, or absent history taking can lead to incorrect diagnoses, unnecessary testing, delays in treatment, and a compromised physician-patient relationship, with the potential for disastrous outcomes. It is thus important to identify the causes of this deficit in essential clinical skills in order to intervene in medical educations.

### The Decline of Clinical Skills

### A multi-factorial phenomenon

Traditionally, bedside teaching has been seen as the ideal clinical teaching modality, in which history taking and physical examination skills can be demonstrated together with professional behavior.  The erosion of bedside teaching and the consequent decline of clinical skills have several causes: excessive reliance on tests, disproportionate time spent at the computer, and limited time for ward bedside rounds and teaching. This is due in part by the fact that attending physicians, residents, and students are often pulled simultaneously in diverse directions. Also, there is the prevailing perception that certain clinical skills are not valued (why spend time in diagnosing a murmur with a stethoscope at the bedside, when an echocardiogram can give us the answer right away?). These, among many other reasons, are possible factors contributing to this phenomenon of declining bedside clinical skills.^17^^,^^18^ Appropriate and comprehensive history taking often tend to be more challenging than conducting a physical examination. Direct observation of students and residents indicates they have difficulty taking a pertinent history and deciding which data of the review of systems, past medical history, and psychosocial/family history are relevant to a specific patient’s case.^19^^-^^22^ As a result, it becomes difficult if not impossible to obtain an accurate account of the symptoms as experienced by the patient. Together with the medical history, the physical examination aids in determining the correct diagnosis and developing the treatment plan.

The content of the history required for appropriate medical evaluation is very variable and will depend on the presenting symptoms, patient concerns, and the past history. Insufficient or ineffective history taking skills may also be linked to inadequate knowledge and lack of clinical exposure. Residents and medical students may need more guidance and training in choosing the most important components of the history and physical exam to best delineate the patient’s problem. Implementing clinical reasoning exercises, during which a medical educator helps small groups of students to work through several of the most common complaints and symptoms physicians are faced with in the daily practice of medicine, may prove useful.^23^^,^^24^ Specifically, learners are asked to think about what elements of the history and physical exam are relevant and why in a given clinical situation. In addition to clinical reasoning exercises, other methods can be used to teach effective history taking skills, such as small group workshops with real or virtual patients, followed by feedback and discussion.^25^ However, clinical reasoning exercises and small group workshops are time-intensive processes which requires the active participation and commitment of many faculty members, who may be already overextended. The systematic implementation of the aforementioned learning techniques poses unique challenges to medical schools. Teaching physical examination skills in a large group setting using computer technology, simulators and audiovisual modalities requires less faculty involvement and is more cost-effective.  With the decline of thorough history-taking and physical examination skills, adequate medical problem-solving skills have decreased as well.^26^^,^^27^

Oversights in history taking and physical examination may lead to delayed diagnosis, unnecessary and potentially harmful treatment, needless testing, escalating medical costs, and potentially life-threatening consequences for patients.  If not addressed, there may over time be a dramatic loss of value associated with the positive patient-provider relationship, which has been shown to produce better health outcomes. This includes taking the time to listen to patients, providing information regarding their illness and subsequent treatment (i.e., promote patient health literacy), taking a proper history and performing an accurate physical exam. These practices can lead to a better rapport with and trust from patients, increased patient and physician satisfaction, patient recall of information, adherence to therapy, and improved patient health outcomes.^28^^-^^30^

### What can medical schools provide?

The underpinnings of this gradual yet steady deterioration of clinical skills such as history taking, and physical examination are complex and represent a significant challenge to academic medical institutions. While a multifactorial phenomenon, the fundamental issue is that often medical schools create an environment which, paradoxically, does not foster the proper instruction of bedside clinical skills. Medical education may sometimes take a backseat to the income-generating endeavors of research and clinical work. Academic institutions’ mission statements traditionally include an emphasis on research, education and clinical service. In reality, many medical schools value funded research as their greatest asset and highly specialized subspecialties which bring in high-revenue patients.  Performance evaluation in academia is biased towards research indicators, while teaching and primary care, both critical to the development of solid clinical skills, are marginalized and all too often at the bottom of the prestige ladder of medical schools. In addition, as academic medical centers have become more dependent on clinical revenue, clinician-educators are currently overloaded with clinical work and are given limited or no protected time for education. Despite their hard work and contributions to the system, clinician-educators receive less financial support, fewer awards and less recognition than they deserve for working with medical students, interns, residents, fellows, providing critical role modeling and molding future generations of physicians. Although clinician-educators are essential to the success and function of modern medical schools, they appear to be profoundly undervalued. All too often, clinician-educators are left in a career limbo, where promotion and advancement reveal to be extremely difficult, causing frustration and lower levels of job satisfaction. A worrisome trend is currently the loss of excellent clinician-educators from academic medical centers.^31^ This ‘exodus’ becomes problematic, generating a vicious cycle with further deterioration of the quality of clinical training and further decline of clinical skills. Medical school administrators and stakeholders might consider re-evaluating their stance on high-quality medical education and invest resources in programs to adequately prepare clinician-educators to teach.  This can be done through providing clinician-educators with true protected time, financial incentives, academic rewards, and a path to promotion and recognition.

### Conflicts of Interest

The authors declare that they have no conflict of interest.
